# Utilization of Tomato Pomace Powder as a Bioactive Ingredient in Semi-Hard Cheese Production: A Study on Nutritional Profile and Sensory Qualities

**DOI:** 10.3390/foods15030542

**Published:** 2026-02-03

**Authors:** Florina Stoica, Roxana Nicoleta Rațu, Iuliana Motrescu, Gabriela Râpeanu, Oana Emilia Constantin, Irina Gabriela Cara, Denis Țopa, Gerard Jităreanu

**Affiliations:** 1 Department of Pedotechnics, Faculty of Agriculture, Iasi University of Life Sciences, 700489 Iasi, Romania; florina.stoica@iuls.ro (F.S.); denis.topa@iuls.ro (D.Ț.); gerard.jitareanu@iuls.ro (G.J.); 2 Department of Food Technologies, Faculty of Agriculture, Iasi University of Life Sciences, 700489 Iasi, Romania; 3 Department of Exact Sciences, Faculty of Horticulture, Iasi University of Life Sciences, 700489 Iasi, Romania; 4 Research Institute for Agriculture and Environment, Iasi University of Life Sciences, 700490 Iasi, Romania; irina.cara@iuls.ro; 5 Department of Food Science, Engineering and Applied Biotechnology, Faculty of Food Science and Engineering, Dunărea de Jos University of Galati, 800201 Galați, Romania; oana.constantin@ugal.ro

**Keywords:** tomato by-products, lycopene, antioxidants, natural ingredients, semi-hard cheese, nutritional enrichment

## Abstract

Tomatoes (*Solanum lycopersicum* L.) are among the most widely consumed and nutritious vegetables globally, being abundant in lycopene, carotenoids, phenolics, organic acids, vitamins, and several other bioactive and health-enhancing compounds. Tomato processing yields a substantial residue known as tomato pomace (TP), primarily composed of peels and seeds, along with a small quantity of pulp. This study investigates the potential of TP powder, rich in dietary fiber, lycopene, polyphenols, and other bioactive compounds, as a natural ingredient in semi-hard cheese. The cheese was enhanced with varying concentrations of TP (5%, 7%), and each variant was assessed for physico-chemicals, sensory properties, minerals, color, phytochemicals, and texture. Cheeses supplemented with TP showed elevated levels of phytochemicals (45.44–82.83 mg GAE/100 g), greater antioxidant capacity (470.25–977.41 µmol TE/g), and higher fiber content (3.62–5.44%), while sensory acceptability remained acceptable at lower inclusion levels but decreased at 7% TP due to slightly bitter aftertaste. Textural analysis showed minimal changes in TP-enriched cheeses, suggesting that TP can be integrated into semi-hard cheese matrices without compromising quality. This study illustrates the feasibility of utilizing TP as an important ingredient in cheese manufacturing, aiding in waste minimization and fostering a circular economy within the food sector. The findings underscore TP’s capacity to enhance dairy products, facilitating innovative and sustainable food solutions that advance health and environmental objectives.

## 1. Introduction

The heightened emphasis on sustainable food systems has sparked substantial interest in valuing agro-industrial by-products and repurposing them as functional food production ingredients to enhance nutritional value and environmental sustainability [1,2].

Among the most widely consumed vegetables globally, tomatoes are abundant in lycopene, phenolics, organic acids, vitamins, and other advantageous compounds. Besides being consumed as a raw vegetable, tomatoes are also utilized in many processed forms, including paste, juice, sauce, puree, and ketchup [3]. Typically, the processing of these items yields a by-product known as tomato pomace (TP), primarily composed of peels and seeds, along with a minor quantity of pulp. Typically, TP constitutes about 3–5% (*w*/*w*) of the utilized raw material [1,4,5].

Tomatoes are predominantly cultivated in China (56.4 million tonnes), the United States (26.1 million tonnes), India (18.4 million tonnes), Turkey (12.6 million tonnes), Egypt (7.94 million tonnes), Italy (6.44 million tonnes), Iran (6.37 million tonnes), Spain (4.67 million tonnes), Brazil (4.17 million tonnes), and Mexico (4.05 million tonnes) [6]. As a seasonal fruit, only a limited portion of the tomato is utilized as a fresh product. Conversely, most tomatoes are processed into products such as juice and paste. TP’s global total yield estimate is 5.4–9.0 × 10^6^ tonnes, although collecting accurate statistics on TP quantity is notably challenging.

The tomato industry produces waste biomass, including TP, consisting of residual peels, cores, culls, pulp, seeds, and discarded tomatoes [7]. The chemical makeup of TP varies based on juice extraction methods; nonetheless, this by-product is typically abundant in readily fermentable fiber and possesses elevated quantities of protein and fat from the remaining seeds [8]. Tomato waste contains a significant amount of bioactives, such as carotenoid compounds (lycopene). TP is a significant agro-food waste in Romania. This particular vegetable waste is widely recognized for its substantial content of polyphenolic chemicals, which possess a significant antioxidant capacity, as well as nutritional fibers [9].

The rising consumer demand for functional products and the necessity to address waste streams from fruit and vegetable processing have led to heightened interest in food science aimed at enhancing their value through product utilization. TP, a by-product of tomato processing, is a lignocellulosic product comprising 33% seeds, 27% peel, and 40% pulp [10]. TP is a substantial source of dietary fiber, comprising up to 60% dietary fiber by dry weight [8]. Moreover, tomato seeds are rich in protein and fat, comprising roughly 35% protein and 25% fat [11]. TP contains a substantial water content (about 80%), rendering it prone to microbial degradation; hence, several drying methods (such as convection or freeze drying) are appropriate for its preservation [12]. Previous studies have demonstrated that TP is a rich source of carotenoids and phenolic compounds, although reported concentrations vary widely depending on extraction method and pretreatment conditions [13,14]. Jamaleddine et al. [13] reported high levels of β-carotene (128.2 mg/g extract) and lycopene (60.1 mg/g extract) in ethyl acetate extracts from TP, along with elevated polyphenol (up to 86.2 mg GAE/g) and flavonoid contents (42.2 mg RE/g). Nour et al. [14] reported relevant levels of polyphenols (1.2 mg GAE/g pomace) and flavonoids (0.4 mg RE/g pomace) using methanolic extraction assisted by sonication.

The disposal or utilization of TP is an inescapable issue and is critically significant for the food business. The improper disposal of TP can lead to spoilage due to its elevated water content and nutrient density, resulting in environmental detriment and resource wastage; conversely, the judicious utilization of TP can convert waste into valuable resources, including lycopene, dietary fibers, and tomato seed oil through biorefinery processes. The systematic disposal of TP constitutes both a resource challenge and an environmental and economic issue. Consequently, food waste management presents an ethical dilemma for society. A strong trend has emerged in recent years toward fortifying dairy products with functional plant ingredients. This is driven by consumer demand for health-promoting, clean-label foods that offer added nutritional benefits without artificial additives. Presently, various improvements have been implemented in the industrial manufacturing of cheese [15], including utilizing waste and by-products as a natural biosource of advantageous added-value compounds. These chemicals can augment nutritional value and mitigate the adverse environmental effects of food waste and by-products [16].

Consequently, research has demonstrated interest in cheese fortification by integrating diverse bioactive components derived from food waste and by-products [17,18]. Among these, TP powder is a promising ingredient due to its rich nutritional profile and potential health benefits [19].

Semi-hard cheese, characterized by its balanced texture and flavour, offers an ideal matrix for incorporating functional ingredients like TP powder [20]. Compared with fresh cheeses, semi-hard cheeses offer improved structural stability during storage and ripening, allowing a more reliable assessment of changes in texture, color, microstructure, and sensory properties following the addition of fiber- and polyphenol-rich ingredients. Furthermore, semi-hard cheeses are widely consumed and technologically robust, making them an appropriate carrier for the development of functional dairy products enriched with agro-industrial by-products [21]. The addition of TP can potentially increase the fiber content and introduce bioactive compounds with antioxidant properties [22,23].

Limited research has been conducted on using TP or powder to enhance the texture and nutritional composition of dairy products [24,25,26]. For example, incorporating tomato powder into cheese boosts its lycopene content, which is valued for its anti-inflammatory and antioxidant properties, while adding tomato by-products enhances the polyphenol profile, further contributing to the product’s health benefits [24].

Carotenoids, particularly lycopene, are highly lipophilic compounds whose solubility and stability are strongly influenced by the fat phase of dairy matrices. In semi-hard cheeses, milk fat can act as a natural solvent for carotenoids, enhancing their dispersion and protecting them against oxidative degradation during processing and storage [27,28,29].

However, despite evidence that tomato powder or tomato-derived ingredients can increase carotenoid content and antioxidant activity in dairy products, limited information is available on how carotenoids from TP interact with the fat–protein network of semi-hard cheeses, particularly in the presence of dietary fiber and polyphenols [21,30,31]. These interactions may affect not only carotenoid stability but also cheese texture, color, and sensory attributes. Consequently, the behavior of TP carotenoids within semi-hard cheese systems remains insufficiently understood, highlighting the need for matrix-specific investigations. Despite the recognized nutritional potential of TP, limited information is available on its interaction with the cheese fat–protein matrix, particularly regarding carotenoid solubility, stability, and their impact on the structural, sensory, and microstructural properties of semi-hard cheeses. The concentrations of TP selected for this study were chosen to balance nutritional enhancement with technological feasibility and sensory acceptability. Moderate inclusion levels were targeted to ensure a measurable increase in phytochemicals, antioxidant activity, and dietary fiber while avoiding excessive adverse effects on texture, color, and flavor that may occur at higher supplementation levels [32,33]. Therefore, two TP concentrations (5% and 7%) were selected to represent a moderate and a relatively high enrichment level, enabling evaluation of both functional benefits and potential sensory limitations in semi-hard cheese systems.

This study aims to assess the effects of TP powder on semi-hard cheese, focusing on nutritional enhancement, phytochemicals, antioxidant activity, sensory attributes, texture, color, and microstructure properties. By examining these aspects, this research aims to demonstrate TP as a sustainable and effective functional ingredient in dairy products, adding value for both the food industry and consumers.

## 2. Materials and Methods

### 2.1. Reagents and Chemicals

The Folin–Ciocâlteu reagent, 6-hydroxy 2,5,7,8 tetramethylchromane-2-carboxylic acid (Trolox), hexane, methanol, ethanol, acetone, [2,20 azinobis (3-ethylbenzothiazoline-6-sulfonic acid) diammonium salt] (ABTS), sodium nitrite, Gallic acid, sodium hydroxide, aluminum chloride, and sodium carbonate were procured from Sigma Aldrich Steinheim (Darmstadt, Germany).

### 2.2. TP Preparation

In August 2024, a quantity of 10 kg of tomatoes at the red stage of ripeness (*Lycopersicon esculentum* var. *Buzău*) was acquired from a nearby supermarket in Iasi, Romania. These tomatoes were thereafter stored at a temperature of 4 °C until they were ready for processing.

The tomatoes were sorted and washed with distilled water before shredding and subsequent compression. The tomato pomace (TP) was obtained by extracting the tomato juice using a kitchen juicer (Bosch MES3500 Philips, Drachten, The Netherlands) in the laboratory. TP consisted of skins, seeds and minimal amounts of pulp remaining from the juicing operations. Before further processing, TP was stored in a deep-freezer cabinet (−20 °C) (Koma, Roermond, The Netherlands). The TP was subjected to freeze-drying (BIOBASE BK-FD10T equipment, Jinan, China) for 45 h at a temperature of −42 °C, with a pressure of 0.10 mBar. The lyophilized was ground in a coffee grinder (Gorenje, Velenje, Slovenia) to obtain TP powder with a moisture content of 8.07%. It was then stored at room temperature in the dark in an airtight glass jar until it was used and analyzed.

### 2.3. Chemical Examinations

The chemical makeup of freeze-dried TP was analyzed. Before chemical analysis, freeze-dried TP was milled into a fine powder using a laboratory grinding mill, and then sieved to obtain a homogeneous particle size fraction (≤500 µm). The powdered TP was thoroughly mixed and stored in airtight containers protected from light at room temperature until analysis.

Moisture, ash, protein, fat, and total dietary fiber were quantified utilizing the procedures outlined by the Association of Official Analytical Chemists [34] namely methods 925.10, 925.51, 925.53, 920.152 (employing a Kjeldahl conversion factor of 6.25), 935.38, and 985.29, respectively. Total sugars were quantified by the Schoorl method [35].

The total dietary fibers of the samples were measured using the method described by AOAC [34]. Carbohydrate was calculated by difference: 100-% (ash + protein + fat + moisture). For each determination, an aliquot of the homogenized powder was accurately weighed and analyzed in triplicate. Moisture content was determined immediately after weighing to minimize the influence of ambient humidity. The remaining analyses (ash, protein, fat, total dietary fiber, and total sugars) were performed on the homogenized TP powder according to the cited AOAC [34] and Schoorl procedures.

### 2.4. Determination of Some Mineral Contents

Mineral contents (Na, K, Ca, Mg, Fe, P, Zn, and Cu) of TP powder were determined according to the methods of AAAC [35] using atomic absorption spectrophotometry (ContrAA 700, Analytik Jena, Jena, Germany).

### 2.5. Color Analysis

The CIELAB attributes (L*, a*, b*) were employed to assess the color of TP powder using a MINOLTA Chroma Meter model CR-410 (Konica Minolta, Osaka, Japan) as outlined in Dag et al. [36].

The calculation of the Chroma and hue angle parameters was performed as follows:

Chroma = $\sqrt{{{(a}^{*})}^{2}+{{(b}^{*})}^{2}}$

Hue angle = arctan(b*/a*) for quadrant I (+a*, +b*).

### 2.6. Extraction of Phytochemicals from TP Powder

The extraction of biologically active compounds from TP was performed using a modified version of the ultrasound-assisted method described by Gheonea (Dima) et al. [37]. In brief, 1 g of TP was mixed with 10 mL of a 70% ethanol solution for the extraction of polyphenols and flavonoids, while another 1 g of TP was combined with 10 mL of a hexane: acetone mixture (3:1, *v*/*v*). The samples were sonicated in a water bath (Elmasonic S 180 H, Elma, Singen, Germany) at 40 °C for 35 min, with a frequency of 40 kHz and a power output of 100 W. The resulting supernatants were collected and centrifuged at 6000 rpm at 10 °C for 10 min. This extraction process was repeated three times, and the combined liquid extracts were concentrated to dryness under reduced pressure at 40 °C using an evaporator (AVC 2-18, Christ, Oxford, UK). The dried extracts were subsequently subjected to phytochemical characterization.

### 2.7. Phytochemical Profile of the TP Extract

#### 2.7.1. Determination of Total Carotenoids and Lycopene

The contents of lycopene and total carotenoids were quantified using a spectrophotometric technique. In a volumetric flask, Souza et al. [38] dissolved 0.2 g of dried extract in 11 mL of an n-hexane: acetone combination (3:1 ratio). The absorbance was quantified at 470 nm and 503 nm. The contents of total carotenoids and lycopene were expressed in mg/100 g dry weight (dw). The quantification of carotenoids was determined using the following Equation (1):

(1) $$Contents\;(mg/100\;g\;dw)\;=\frac{A\times Mw\times Df}{Ma\times L\times m}$$

where A—absorbance of the sample, Mw—molecular weight, Df—sample dilution rate, Ma—molar absorptivity for lycopene in n-hexane (3450 L mol^−1^ cm^−1^), total carotenoids in n-hexane (2500 L mol^−1^ cm^−1^), m—mass/weight of concentrated extract, and L—cell diameter of the spectrophotometer (1 cm).

#### 2.7.2. Determination of Total Flavonoid Content

The total flavonoid content of the extract was quantified using the aluminum chloride spectrophotometric method [39]. A solution was prepared by blending 250 µL of the extract with 2 mL of distilled water and 75 µL of 5% sodium nitrite (NaNO_2_). After 5 min, 150 µL of aluminium chloride (AlCl_3_) was added to the liquid. The combination was measured with a UV-Vis spectrophotometer (Analytik Jena Specord 210 Plus, Jena) at a wavelength of 510 nm, following the addition of 500 µL of a 1 M sodium hydroxide (NaOH) solution 6 min later. The results were expressed as milligrams of catechin equivalents (mg CE/g dw) using a calibration curve for catechin.

#### 2.7.3. Determination of Total Polyphenol Content

The total polyphenol content of the extract was quantified using the Folin–Ciocâlteu method through spectrophotometric analysis [39]. A total volume of 200 µL of the extract was well mixed with 15.8 mL of distilled water and 1 mL of Folin–Ciocâlteu reagent. After 10 min, the mixture had been supplemented with 3 mL of 20% Na_2_CO_3_. The resultant mixture was stored at room temperature in a dark setting for 60 min before quantification with a UV-Vis spectrophotometer (Analytik Jena Specord 210 Plus, Jena) at a wavelength of 765 nm. The data were calibrated via a gallic acid calibration curve and expressed as milligrams of gallic acid equivalents (mg GAE/g dw).

#### 2.7.4. Determination of the ABTS+ Scavenging Activity

The ABTS+ radical technique was employed as outlined by Xu et al. [40]. The generation of the ABTS radical cation (ABTS+) involved the reaction of equal volumes of a 7 mM ABTS stock solution with 2.45 mM K_2_S_2_O_8_. The resultant mixture was subsequently allowed to stand in darkness for 16 h before its application. Additionally, 1 mL ABTS+ solution was diluted with 35 mL of ethanol to get an absorbance value of 0.700 ± 0.02 (A0) at a wavelength of 734 nm. A volume of 1800 µL of the ABTS+ solution and 200 µL of the extract solution (Af) was left to react for 2 h in a dark environment before measuring its absorbance at 734 nm. The antioxidant activity of the extract was quantified in µmol Trolox Equivalent (TE)/g dw according to the calibration curve. The radical scavenging activity was also reported as the percentage of inhibition according to Equation (2):

(2) $$ABTS\;+\;scavenging\;activity\;(\%)=\frac{A0-Af}{A0}\times 100$$

### 2.8. HPLC Investigation of the Carotenoids from the TP Extract

The separation of carotenoids from the extract obtained from TP was carried out in a concentration gradient using acetonitrile 90% (*v*/*v*) (solvent A) and ethyl acetate 100% (solvent B) as described by Gavil (Rațu) et al. [41] using a Thermo Finnigan Surveyor HPLC system with a DAD UV–visible detector (Finnigan Surveyor LC, Thermo Scientific, Waltham, MA, USA) The carotenoid compounds from the TP were analyzed at 450 nm on a Lichrosorb RP-18 (5 μm) Hibar RT 125–4 column. The identification and quantification of the primary carotenoids were conducted using the calibration curves of the existing standards.

### 2.9. Fourier Transform Infrared Spectroscopy (FTIR) Spectra

FTIR spectra for the molecular characterization of TP powder were acquired in triplicate (n = 3) for each sample using an Interspec 200-X FTIR spectrometer (Interspectrum, Tartu, Estonia). Spectra were recorded over the 500–4000 cm^−1^ range, with a resolution of 1 cm^−1^ [42].

### 2.10. Preparation of TP-Supplemented Semi-Hard Cheese

The functionality of enhanced semi-hard cheese was evaluated by incorporating TP at two inclusion levels (5% and 7%-CTP5%, CTP7%). A control sample (CC) was produced without TP addition. The selected concentrations were established based on preliminary trials and positive sensory evaluations performed by the panellists, which indicated optimal visual appearance, texture, flavour, and colour at these levels. TP powder was added at 5% and 7% (*w*/*w*), calculated relative to the raw milk mass at the batch formulation stage, prior to coagulation. The powder was uniformly dispersed in the milk before rennet addition to ensure homogenous distribution. These inclusion levels were chosen in accordance with literature reports indicating that lower concentrations may provide limited functional benefits, whereas higher concentrations can negatively affect cheese texture, colour intensity, and sensory acceptability due to the high dietary fibre and polyphenol content of TP. The selected levels therefore enabled the identification of a threshold at which nutritional and antioxidant enhancement could be achieved without compromising technological feasibility or sensory quality.

**Figure 1:**
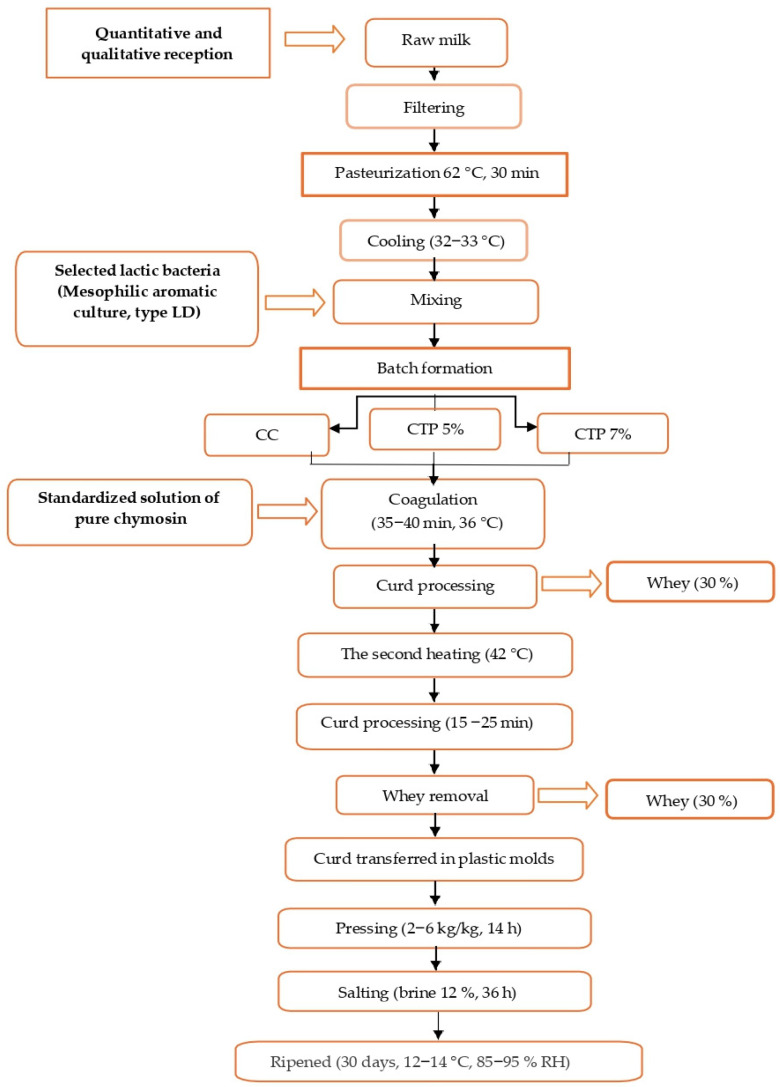
The technological flow of semi-hard cheese manufacturing.

Figure 1 presents the technological flow diagram for semi-hard cheese production [43]. The process commenced with the quantitative and qualitative assessment of raw milk. After filtration, the milk was pasteurised at 62 °C for 30 min in a processing vat and subsequently cooled to 32–33 °C. Starter cultures were then added, consisting of a mesophilic aromatic culture (*Lactococcus lactis* subsp. *cremoris*, *Lactococcus lactis* subsp. *lactis*, *Lactococcus lactis* subsp. *lactis biovar diacetylactis*, and *Leuconostoc* spp.), namely Ld-Fd-DVS Flora Danica (CHR Hansen, Hørsholm, Denmark). The inoculation rate was 2 g per 100 L of milk, followed by a resting period of 30 min to allow culture activation.

Milk coagulation was carried out at 35–36 °C using CHY-MAX^®^ M liquid rennet at a dosage of 10 mL per 100 L of milk, in accordance with the manufacturer’s recommendations for semi-hard cheese production. This rennet was selected due to its high specificity towards κ-casein, resulting in efficient coagulation and the formation of a firm and uniform curd.

After partial curd formation, approximately 30% of the whey was removed, with total coagulation time ranging between 35 and 40 min. A second heating step was subsequently applied at 42 °C, during which the curd was cooked for 10–15 min, until adequate dehydration of the curd grains was achieved. The curd was then pressed at 2 kg kg^−1^ for 14 h in plastic moulds containing 2 kg of curd. During the first 8 h of pressing, the moulds were turned every 10 min. The process concluded with salting in 12% (*w*/*v*) brine, followed by ripening for 30 days at 12–14 °C under controlled relative humidity conditions (85–95%).

### 2.11. Characterization of the Physicochemical Properties of the TP-Supplemented Semi-Hard Samples

The AOAC, 2000 techniques [35] were employed to assess the physicochemical properties of the supplemented samples, including their moisture and dry matter contents, pH, protein, fat, carbohydrates, fiber, ash, and salt. The energetic value of the samples was estimated according to the methodology outlined by Postolache et al. [39] based on the proximate analysis results. Energy value = (% crude protein × 4) + (% crude fat × 9) + (% carbohydrates × 4) + (% crude fiber × 2).

### 2.12. Characterization of Phytochemicals and Storage Stability of the TP-Supplemented Semi-Hard Samples

Using the methods previously outlined in Section 2.7. Phytochemical profile of the TP extract, were assessed the total carotenoids, lycopene, flavonoids, phenolic content, and antioxidant activity of semi-hard cheese enriched with TP.

Before phytochemical analysis, cheese samples were prepared as follows: at each sampling time (day 0 and day 30), cheese was crushed via a laboratory grinder to obtain a homogeneous material and immediately analysed or stored at 4 °C until extraction. For each determination, an accurately weighed portion of crushed cheese was used.

The samples were housed in light-resistant glass containers and stored at 4 °C for 30 days to assess storage stability. Consequently, variations in the phytochemical content were observed. The contents of total carotenoids, lycopene, flavonoids, and polyphenols were expressed in mg/100 g fresh weight.

### 2.13. Color Evaluation of TP-Supplemented Semi-Hard Samples

The CIELAB parameters (L*, a*, b*) were utilized to evaluate the color of TP and TP-enriched samples, as described in Dag et al. [36]. Before analysis, freeze-dried TP powder was placed in a uniform layer in a glass sample holder to obtain a smooth, opaque surface. Cheese samples were cut into slices of uniform thickness and equilibrated to room temperature before measurement. Color parameters (L*, a*, b*) were recorded at multiple points on the sample surface, and mean values were calculated.

The estimation of the Chroma (C*), hue angle (H*), and total color difference (ΔE) parameters was done as follows:

Chroma (Chroma = $\sqrt{{{(a}^{*})}^{2}+{{(b}^{*})}^{2}}),$

Hue angle (H*) = arctan(b*/a*) was determined for quadrant I (+a*, +b*), H* = 180 + arctan(b*/a*) for quadrant II (−a*, +b*),

ΔE = $\sqrt{{{(L}^{*}-L_{0})}^{2}+{{(a}^{*}-a_{0})}^{2}+{{(b}^{*}-b_{0})}^{2},}$ L_0_, a_0_, b_0_ = blank value of each sample.

### 2.14. Textural Parameters of TP-Supplemented Semi-Hard Samples

The texture of the TP-supplemented semi-hard cheese samples was evaluated using the Texture Profile Analysis method, employing a Brookfield CT3 Texture Analyser (Brookfield Engineering Laboratories, INC. Middleboro, Massachusetts, USA). The cheese samples (in the form of cylinders, 7 mm in length and 12 mm in depth) were subjected to compression to 50% of their original height using a cylindrical probe (TA11/1000) with a 25.4 mm diameter, a 1000 g load cell, and a cross-head speed of 2.0 mm/s. Two compression cycles were performed, and the data were collected using TexturePro CT V1.6 software. Texture profile analysis was performed on samples obtained from two independent cheese batches per formulation. From each batch, multiple cylindrical specimens were prepared and analyzed, with each specimen tested in duplicate. The reported values therefore represent the mean of several measurements per formulation. Before testing, they were equilibrated to ambient temperature (about 20 °C).

### 2.15. Minerals Evaluation of TP-Supplemented Semi-Hard Samples

A flame atomizer system was employed to quantify the mineral content of the samples via atomic absorption spectrometry (ContrAA 700, Analytik Jena, Jena). Measurements of mineral content (calcium, phosphorus, magnesium, potassium, zinc, iron, copper, and sodium) in the experimental cheese samples were performed using a MiniWAVE Microwave digestion equipment (SCP Science, Baie-d’Urfé, QC, Canada) with a 50 mL Teflon vial. One gram of the homogenized sample was weighed and transferred into a Teflon vial. The sample was subsequently digested with a mixture of nitric acid (HNO_3_) and hydrochloric acid (HCl) in an 8:2 ratio. The digestion process was performed under defined parameters: a temperature of 180 °C, a duration of 50 min, and a microwave power of 1000 W. After the cooling process, the sample was carefully placed into a 50 mL volumetric flask and then diluted with ultrapure water to achieve the necessary concentration. A blank sample was incorporated in every digestion run, and each sample was prepared in triplicate. The data were presented in milligrams per 100 g dw.

### 2.16. Morphology

A scanning electron microscope (SEM) (Quanta 450, FEI, Thermo Fisher Scientific, Hillsboro, OR, USA) equipped with an energy dispersive X-ray detector (EDS) (EDAX, AMETEK Inc., Berwyn, PA, USA) was employed to elucidate the morphological characteristics of the experimental samples. The EDS spectral analysis was conducted using the TEAM version V4.1 equipment manufactured by EDAX Inc. An AlCu sample composed of copper foil on an aluminum grid was used for calibration before the study. The samples were examined in a low-vacuum setting with a pressure of approximately 6.1 × 10^−4^ Pa. The electrons were accelerated at a voltage of 15 kV, and the pictures were analyzed at a magnification of 500× (100 μm).

### 2.17. Sensory Evaluation of TP-Supplemented Semi-Hard Samples

Sensory evaluation was conducted as a preliminary acceptability test using a semi-trained panel composed of 25 volunteers (students and academic staff) from the Faculty of Agriculture, University of Life Sciences in Iași, Romania. Panelists were recruited based on the following criteria: regular consumption of cheese (at least once per week), absence of self-reported sensory impairments (taste, smell, or color vision deficiencies), and willingness to participate voluntarily. Prior to evaluation, panelists received a brief orientation session explaining the study objectives, evaluation procedures, use of the hedonic scale, and definitions of the assessed attributes (appearance, color, texture, flavor, aftertaste, and overall acceptability). Although panelists were not formally trained sensory experts, they were familiar with dairy products and basic sensory assessment concepts through academic or professional exposure. The sensory analysis was therefore designed to provide indicative information on acceptability trends rather than representative consumer preference data.

The participants were told to assess 11 descriptors: appearance, section appearance, color, flavor, acid odor, creamy odor, fruity, bitter taste, salty taste, acid taste, aftertaste, firmness, chewiness, and overall quality. The panelists were instructed to evaluate each sensory attribute of seven scores utilizing the Hedonic scale, which ranges from 1 (representing extreme dislike) to 7 (representing extreme liking). The TP-enriched cheeses were randomly assigned three-digit codes, cut into cubes, and served on a plate next to the control cheese. Mineral water and unsalted crackers served as palette cleansers between tastings. The analysis was performed according to the specifications given in ISO 858929 [44] and Bérodier et al. [45].

### 2.18. Statistical Analysis

Statistical analysis was performed using Minitab^®^ 19 software (Minitab LLC, State College, PA, USA). A one-way analysis of variance (ANOVA) was applied to evaluate the effect of cheese formulation (control, 5% TP, and 7% TP) on each measured parameter, including physicochemical properties, phytochemical content, antioxidant activity, texture, color, and sensory attributes. When storage was considered, comparisons among formulations were performed within each storage time point. Mean comparisons were carried out using Tukey’s post hoc test at a significance level of *p* < 0.05. All results are expressed as mean ± standard deviation, based on at least two independent determinations for each measurement. Principal Components Analysis (PCA) was conducted on the descriptive sensory data using the XLSTAT program (version 2024.3, Addinsoft, Paris, France).

## 3. Results and Discussion

### 3.1. Global Characterisation of TP

The concentration of biologically active chemicals in TP extracts was analyzed using spectrophotometric techniques. Table 1 presents the phytochemical content (polyphenols, flavonoids, carotenoids, and antioxidant activity), physicochemical, color and mineral properties of TP powder. Consequently, the extract presented total carotenoid content of 1030.81 ± 0.53 µg/g dw, a lycopene content of 561.25 ± 0.09 µg/g dw, a total flavonoid content of 112.18 ± 1.38 mg CE/100 g dw, and a total polyphenolic content of 344.38 ± 2.06 mg GAE/100 g dw. The extract demonstrated an antioxidant activity of 1431.60 ± 5.84 µmol TE/g dw and an inhibition rate of 94.58 ± 0.39%. The plant’s antioxidant capacity was associated with its elevated phenolic compounds, especially flavonoids, the primary secondary metabolites.

The results that were obtained align with the data presented in other studies.

Yilmaz, Kumcuoglu, and Tavman [46] suggested that, with the help of ultrasound (90 W), the amount of lycopene extracted from TP using a mixture of hexane-acetone-ethanol (2:1:1) increased from 52.21 mg/kg to 70.10 mg/kg. Silva et al. [47] conducted an ultrasound-assisted extraction of lycopene from TP using a solvent mixture composed of ethyl lactate and ethyl acetate. Under optimized conditions—63.4 °C, 30% (*v*/*v*) ethyl acetate in the solvent mixture, a solvent-to-sample ratio of 100 mL/g, and an extraction time of 20 min, a high lycopene yield of 1334.8 ± 83.9 μg/g was achieved efficiently, at a relatively mild temperature and within a short duration.

Kehili et al. [48] used the hexane maceration technique to extract lycopene from tomato skin, reporting a lycopene concentration of 532 ± 23.11 μg/g dw.

Nagarajan et al. [49] revealed a total carotenoid concentration of 9.37–10.81 mg/100 g for wet samples. Vagi et al. [50] demonstrated that the lycopene content in the carotenoid-rich extract ranged from 70% to 90%, establishing lycopene as the most significant compound in TP, with a concentration of 31.40 mg/100 g. A study indicated that both drying methods of TP and extraction methodologies affect the yield of lycopene, which may range from 5.66 to 59.66 μg/g [51]. The extraction process influences the total phenolic content of TP. The total phenolic content for the microwave-assisted extraction approach ranged from 2.85 to 17.87 mg GAE/g, while for the ultrasound-assisted extraction, it varied between 1.81 and 18.65 mg GAE/g [52].

The TP displayed color characteristics of 48.43 for the L* value, 26.82 for the a* value, and 36.28 for the b* value. The color of TP is mainly due to the lycopene pigment. The color indices indicate that the powder is in the first quadrant (+a*, +b*).

The chemical composition of TP is shown in Table 1. The chemical composition of TP reflects its origin as a processing by-product primarily composed of peels, pulp and seeds, which differ substantially from the edible pulp of raw tomatoes. While fresh tomatoes typically contain high moisture levels (above 90%) and relatively low concentrations of fiber and minerals, TP represents a concentrated fraction in which water-soluble components are largely removed during juice or paste production, resulting in an enrichment of structurally bound constituents. In the present study, TP showed high levels of dietary fiber (46.10% dw), moisture (10.52% dw), carbohydrates (67.09% dw), protein (11.45% dw), fat (6.14% dw), and ash (4.80% dw), confirming its higher nutrient density compared with whole tomatoes. Also, Silva et al. [53] showed that the composition of TP includes moisture (62.27–70.14%), protein (16.81–23.25 g/100 g), fat (11.17–16.73 g/100 g), dietary fiber (48.62–53.97 g/100 g), and ash (3.33–4.02 g/100 g), respectively.

The mineral composition of TP was analyzed using an atomic absorption spectrophotometer and was presented in Table 1. Similarly, the mineral profile of TP demonstrates its added value relative to raw tomatoes, as peels, pulp, and seeds are known to retain higher concentrations of minerals such as calcium, magnesium, potassium, and iron. The elevated levels of Mg and Ca observed in this study, followed by K, Na, P, Fe, Zn, and Cu, are consistent with literature reports according to the study by Chabi et al. [54], indicating that TP is a significant mineral source due to the preferential localization of these elements in non-pulp tissues. The degree of repining [55] and agricultural conditions [56] affect the mineral content of tomatoes. Thus, TP can be considered a nutritionally concentrated derivative of the raw tomato, supporting its use in the development of value-added functional foods.

### 3.2. HPLC Investigation of Carotenoids from the TP Extract

**Figure 2:**
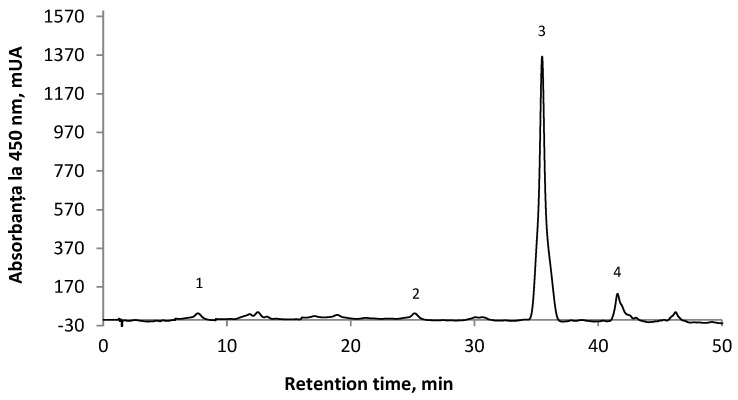
HPLC profile of TP carotenoids: 1—zeaxanthin; 2—lycoxanthin; 3—lycopene and 4—β-carotene.

HPLC analysis of TP extract carotenoids comprises extraction, separation, identification, and quantification of primary carotenoids such as lycopene, β-carotene, lycoxanthin, and zeaxanthin, as presented in Figure 2. The predominant carotenoid identified in the tomato peel was lycopene, followed by β-carotene. The results agree with those obtained by García-Valverde et al. [57] and Ferrando et al. [58]. Moreover, Huang et al. [59] established an efficient and accurate LC-DAD technique for quantifying lutein, β-carotene, and lycopene in tomatoes and their derivatives.

Also, in a study by Li [60] using a rapid and sensitive UPLC analysis on a 1.7 μm C18 column separated and identified all-trans-lutein, lycopene, β-carotene, and their 22 cis-isomers in 20 tomato varieties.

### 3.3. FTIR Spectra

FTIR spectra revealed the presence of bioactive compounds in TP powder.

**Figure 3:**
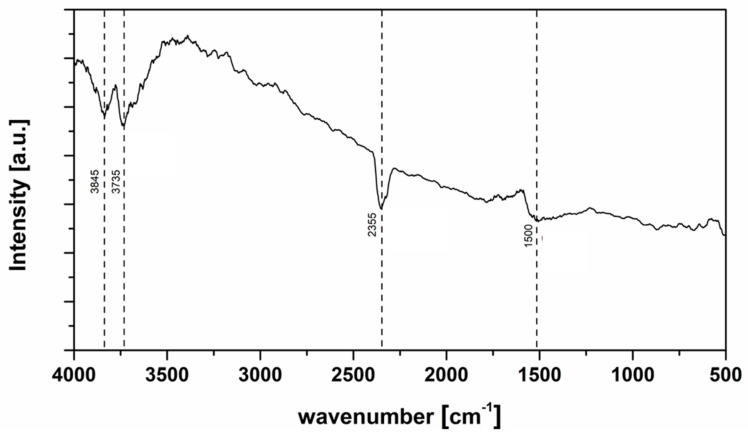
FTIR spectra of the TP powder.

The FTIR spectrum of TP powder (Figure 3) reveals key functional groups that indicate the presence of various bioactive compounds. The FTIR spectral features of TP powder are consistent with its chemical and phytochemical composition previously discussed in Section 3.1 (Table 1). A broad and intense absorption band around 3285 cm^−1^ corresponds to O–H stretching vibrations, characteristic of alcohols and phenolic compounds, suggesting a high content of hydroxyl-rich molecules such as polyphenols and flavonoids (344.38 mg GAE/100 g dw and 112.18 mg CE/100 g dw, respectively). This broadness also reflects strong hydrogen bonding, common in moisture-rich or hydrophilic plant matrices. A broad band observed between 3900 and 3500 cm^−1^ indicated the presence of O–H and C-H groups. Additionally, the peak at 3735 cm^−1^, corresponding to –OH stretching vibrations, was attributed to hydrogen-bonded hydroxyl groups found in polysaccharides or polyphenols [61]. The peak at approximately 2935 cm^−1^ is associated with C–H stretching vibrations of aliphatic –CH_2_ and –CH_3_ groups, indicating the presence of lipids and saturated fatty acids, likely originating from tomato seeds and skin (6.14% fat). The absorption peaks in the range 2500–2000 cm^−1^ correspond to the stretching of C=O in the carboxyl groups (–COOH) which can be attributed to the bonds present in β-carotene [62].

A shoulder near 2856 cm^−1^ may correspond to symmetric C–H stretches or weak C=O signals from carboxylic acids or esters, although no strong, well-defined carbonyl peak is evident in the typical 1740–1650 cm^−1^ range. At 1595 cm^−1^, a clear absorption band is observed, which can be attributed to C=C stretching in aromatic rings, pointing to the presence of aromatic structures like flavonoids and other polyphenolic compounds. The bands detected in the 1550–1250 cm^−1^ region of the FT-IR spectrum of TP indicate C=C stretching vibrations, which are characteristic of pectin fractions and polysaccharides [63], in agreement with the high dietary fiber content (46.10%).

While not distinctly labelled in the figure, the fingerprint region between 1050–1250 cm^−1^ likely contains overlapping C–O stretching vibrations from carboxylic acids, esters, or ether groups associated with pectins, cellulose, or organic acids. Overall, the spectrum highlights the rich composition of TP in phenolic, aliphatic, and acid-derived functional groups, supporting its potential use in food and nutraceutical applications. The FTIR profile corroborates the compositional data, confirming that TP is a complex matrix rich in phenolics, carotenoids, lipids, and structural polysaccharides, which underpins its functional behavior when incorporated into semi-hard cheese systems.

### 3.4. Characterization of Phytochemicals of TP-Supplemented Cheeses and Storage Stability of the Samples

Table 2 presents TP-enriched cheeses’ bioactive compounds and antioxidant activity at various storage intervals. Significant differences (*p* < 0.05) were observed in the phytochemical properties and antioxidant activity across all semi-hard cheese samples. Incorporating TP (5–7%) into semi-hard cheese enhanced total carotenoids, lycopene, flavonoids, polyphenols, and antioxidant activity.

Increasing TP supplementation from 5% to 7% significantly enhanced the levels of carotenoids, lycopene, flavonoids, and polyphenols in the enriched cheese samples compared to the control. The addition of tomato powder notably boosted the ABTS scavenging activity of the cheeses, with the most pronounced increase observed in CPP7%. This improvement is likely due to the high antioxidant capacity of TP, which is a rich source of total phenolics. These findings align with previous studies showing that TP exhibits antioxidant properties, primarily attributed to its bioactive and functional compounds, particularly phenolics and flavonoids [11,64,65].

Likewise, Roila et al. [66] examined the application of a polyphenolic extract sourced from olive oil by-products to enhance the functionality of “Fior di latte” cheese. The extract was included in two cheese batches at 250 and 500 μg/mL doses. The extract markedly elevated the overall phenolic content of the cheese, enhancing its functional value and health-promoting attributes. The pigments for cream cheese were assessed utilizing extracts from the fruit of Sea Buckthorn (*Hippophae rhamnoides* L.) [67]. The principal measurable pigments and polyphenols identified in the fruit extracts were carotenoids (8.27 mg/L total carotenoids) and total polyphenols (1842.86 mg/100 g). Marchiani et al. [68] noted that incorporating grape pomace powders (Barbera, Chardonnay) into semi-hard cheeses (Italian Toma-like) at a concentration of 1.6% (*w/w*) markedly enhanced phenolic content, radical scavenging activity, and antioxidant activity.

Incorporating tomato powder in processed cheese formulations enhanced the final product’s functional characteristics, elevated antioxidant activity, and improved flavor attributes [69]. Lucera et al. [24] discovered that cheese fortified with grape pomace powder demonstrated a markedly greater enhancement in total phenolic content, flavonoids, and antioxidant activity than the control cheese.

Over a 30-day storage period, the phytochemical concentration rose, hence influencing the antioxidant activity. Over time, the total carotenoids in the TP-supplemented samples exhibited a small rise. The total carotenoids in TP-enriched samples ranged from 90.05 to 140.41 mg/100 g fresh weight initially and increased to 120.07 to 180.37 mg/100 g fresh weight after 30 days of storage. The total polyphenols exhibited a substantial increase (*p* < 0.05). When in storage. The total polyphenol content was 123.66 ± 2.39 mg GAE/100 g fresh weight of CTP7 cheese on the initial day and increased to 155.21 ± 2.74 mg GAE/100 g fresh weight of CTP7 cheese on the final day. The elevated polyphenol values may be attributed to the presence of milk-derived compounds and the non-selectivity of the Folin–Ciocalteau reagent used in the analysis. This reagent reacts with phenols and other reducing substances, including carotenoids, amino acids, sugars, and vitamin C, potentially reducing analytical accuracy [70]. The elevation of polyphenols in the cheeses over the 30-day storage period resulted in a significant increase in their antioxidant activity (ABTS), also (*p* < 0.05). Our findings are consistent with those of Mahajan, Bhat, and Kumar [71], who reported improved oxidative stability in buffalo cheese “Kalari” enriched with pomegranate rind extract (2000 mg/100 mL); however, unlike their use of a soluble extract in a fresh cheese, the present study employed a solid, fiber-rich TP in a semi-hard cheese matrix, which may modulate antioxidant behavior through matrix interactions rather than direct preservative action.

The apparent increase in total polyphenols, carotenoids, and antioxidant activity observed during the 30-day storage period should not be interpreted as de novo chemical synthesis of these compounds within the cheese matrix. Instead, this trend likely reflects a combination of concentration effects caused by enhanced extractability of tomato-derived phytochemicals as the cheese structure evolves. Also, biochemical and structural changes occurring during ripening, such as proteolysis and fat rearrangement, may improve the accessibility of polyphenols and carotenoids previously entrapped within the protein–fat matrix, thereby increasing their extractability during analysis [72,73]. Furthermore, the Folin–Ciocalteu assay used for total polyphenol determination is known to be non-specific and responds to a wide range of reducing substances, including peptides, amino acids, and other ripening-derived compounds. The formation of these reducing components during storage can contribute to higher apparent “polyphenol” values without representing a true increase in phenolic content [74].

In lipid-containing matrices such as semi-hard cheese, antioxidant activity should also be interpreted in relation to lipid oxidation stability. Indicators such as peroxide value (PV), which reflects primary lipid oxidation products, and thiobarbituric acid reactive substances (TBARS), associated with secondary oxidation products, are widely used to assess oxidative deterioration in dairy products. Previous studies have shown that the incorporation of plant-derived antioxidants rich in polyphenols and carotenoids can significantly reduce PV and TBARS values during cheese storage, indicating delayed lipid oxidation [75].

Although PV and TBARS were not directly measured in the present study, the observed increase in antioxidant activity (ABTS) in TP-supplemented cheeses suggests an enhanced capacity to scavenge free radicals and potentially inhibit lipid oxidation. This interpretation is consistent with earlier reports in which tomato-derived ingredients or other fruit by-products added to dairy matrices resulted in lower lipid oxidation indices during storage [76,77]. Therefore, the elevated antioxidant activity observed in TP-enriched cheeses likely contributes to improved oxidative stability of the lipid fraction, supporting the functional role of TP beyond phytochemical enrichment. Nevertheless, future studies incorporating direct lipid oxidation measurements (PV, TBARS) would provide additional confirmation of the protective effect of TP against oxidative degradation in ripened cheeses.

The data in Table 2 confirm that enriching cheese with TP enhances its quality by increasing carotenoid and polyphenol concentrations, which boosts antioxidant activity. This study highlights TP as a natural alternative to synthetic colorants and antioxidants.

### 3.5. Physico-Chemical Characterization of TP-Supplemented Cheese Samples

Table 3 outlines the physicochemical properties of semi-hard cheeses incorporating different proportions of TP (5% and 7%) compared to the control cheese.

Incorporating TP into cheese significantly enhanced its chemical composition relative to the control sample. A significant change (*p* < 0.05) was observed in the proximate composition of the semi-hard cheese samples with and without TP addition. Including TP leads to substantial changes (*p* < 0.05) in moisture, fat, carbs, protein, fiber, ash, energy value, and dry matter content. No notable variations in pH and salt concentration were detected among the samples.

Incorporating TP into cheese reduced carbohydrate and moisture contents while increasing protein, fat, fiber, ash, and dry matter, with these changes becoming more pronounced as the level of TP addition increased. The obtained results can be ascribed to the TP’s high concentrations of dietary fiber and crude protein [78].

Increasing the proportion of TP significantly elevated the total solids and total protein content in semi-hard cheese (*p* < 0.05). Concerning the total protein content (Table 3), assessed on a dry matter basis, dietary supplementation with TP elicited significant changes (*p* < 0.05). The protein content measured 14.90%, 15.29%, and 16.43% in the control, CTP5, and CTP7 cheeses, respectively, indicating a progressive increase with adding tomato powder. Moreover, incorporating 7% tomato powder during cheese production had a statistically significant effect (*p* < 0.05) on fiber content, with CTP7 recording the highest fiber value at 5.44%.

The pH value slightly decreased compared to CC, attaining 5.06 ± 0.10 for the CTP7 sample. Incorporating TP powder elevated the ash content, resulting in an average value of 2.62 ± 0.15% for the CTP7 sample. The salt content slightly decreased for the enriched cheeses compared to the control sample. The energetic value increased with the TP addition but was within the range of the conventional product.

Lucera et al. [24] indicated that using flour derived from artichoke external leaves at a 5% (*w*/*w*) concentration enhanced the fiber content of cheese made from pasteurized skimmed cow milk (0.1% fat) [16]. Italian Vastedda cheese, enhanced with 1% (*w*/*w*) red grape pomace powder and manufactured using four lactic acid bacteria and ovine milk, reduced fat content and increased protein content [79]. Rehal and Kaur [80] conducted a study assessing the impact of TPP incorporation in Bhujia, an Indian snack, at different concentrations (0–12.5%). Results indicated that TPP addition of up to 5% significantly enhanced the product’s acceptability regarding sensory attributes. The product yields were enhanced relative to the control sample, which lacked any addition of TP powder. The compositional analysis of the product indicated it is a rich source of fiber (3.40%), fat (20.8%), protein (16.52%), antioxidant activity (38.5%), and energy value (485.32 kcal/100 g).

### 3.6. Color Evaluation of TP-Supplemented Cheese Samples

Considering that the color of semi-hard cheese significantly influences customer acceptance, it is essential to assess it thoroughly. The color parameters of TP-enriched cheeses were evaluated initially and after storage, with the results detailed in Table 4. A significant difference (*p* < 0.05) was observed between the unsupplemented cheese and the samples enriched with TP.

The dietary addition significantly affected the brightness (L*) of the cheese, with lower values seen in CTP5 and CTP7 cheese compared to control (*p* < 0.05). The a* and b* values, which indicate redness/greenness and yellowness/blueness, respectively, were higher in TP-enriched samples compared to the control. Notably, adding TP increased both a* and b* values in the cheeses. Adding TP to the food product generally reduces lightness, while redness and yellowness markedly rise [81]. The yellowness value (b*) of TP-enriched samples was significantly higher (*p* < 0.05) than that of the control. Adding TP increased b* values, giving the cheese a more pronounced yellowish hue. This effect is likely attributed to the high carotenoid content present in TP [64]. Similarly, incorporating grape pomace powder in ovine stretched cheese significantly modified exterior and internal color metrics [79]. The indices observed in control cheeses corresponded with those documented for ovine-stretched cheeses by Todaro et al. [82].

During the storage time, L* values decrease, and a* and b* values increase.

The ripening process influenced the yellow chromaticity (b*). The fluctuations in the chromatic coordinates b* throughout ripening were solely due to the aging effect, likely resulting from pigment concentration and oxidation. All TP-supplemented samples obtained during ripening exhibited a notable decrease in brightness without a corresponding increase in yellowness.

The CIELCH parameters include the Chroma value, representing color saturation, and the H* value, indicating the hue angle and chromaticity or color tone. The color saturation (Chroma) values differed between the control and TP-enriched samples, aligning with the b* color coordinate. Additionally, the Chroma values increased with TP addition and after storage, with the highest Chroma observed in the CTP7% cheese.

The color tone (H*) values ranged from 178.54 to 178.52 for the control cheese for quadrant II(−a*, +b*) and 1.03 to 1.02 for the CTP7 sample for quadrant I (+a*, +b*) during storage for 30 days. The H* decreased for the TP-enriched samples [36].

The overall color change of the samples ranged from 31.59 ± 0.04 to 37.66 ± 0.05, induced by the addition of TP. Augmenting the TP content resulted in an elevation of the ΔE values. A primary objective for calculating total color difference (ΔE) is to ascertain whether the color disparity between samples with additional TP powder and the control is discernible to the human eye. Visual color differences can be classified by total color difference into the following categories: undetectable (0–0.5), moderately noticeable (0.5–1.5), discernible (1.5–3.0), definitely visible (3.0–6.0), and substantially different (6.0–12.0). The impact of TP on the color of cheese samples is evident, which can be attributed to the natural color of TP [65].

The ΔE values above 30 observed in TP-enriched cheeses reflect a pronounced shift toward orange–red hues, which is consistent with the high carotenoid content of TP. Sensory analysis confirmed that panelists clearly perceived these color changes; however, this did not negatively affect overall acceptability at the tested moderated inclusion levels. On the contrary, the color was described as characteristic and coherent with the presence of tomato-derived ingredients. The CTP7 sample (7% TP) was characterized by an intense, vivid color; however, some panelists perceived its flavor as overly strong, with a slight bitter aftertaste. Overall acceptability scores indicated that the semi-hard cheese formulated with 5% TP was better accepted.

Consumer acceptance of such color changes is strongly dependent on product positioning and expectation. In the context of a functional or specialty semi-hard cheese intentionally enriched with TP and marketed as a vegetable-enhanced or gourmet product, an orange/red coloration can be considered desirable and indicative of added value rather than a defect. Similar acceptance trends have been reported for cheeses and dairy products fortified with vegetable- or fruit-derived ingredients, where distinct color changes are expected and aligned with flavor perception [31,71].

### 3.7. Textural Properties of TP-Supplemented Cheese Samples

Bourne [83] asserts that texture constitutes the structure of food and is acknowledged as a critical determinant of product acceptance that influences customer opinion. Table 5 illustrates the effect of TP addition on the textural profile characteristics (hardness, adhesiveness, cohesiveness, gumminess, elasticity, and chewiness) of cheese samples at the initial moment and throughout a 30-day storage duration.

The results revealed that the inclusion of TP significantly influenced the textural properties of the cheese samples. Moreover, the cheese containing the highest concentration of TP demonstrated the most notable changes in texture compared to the control cheese A texture profile analysis test was conducted to assess various textural parameters, which are consistently linked to the water content and the fat and protein composition that define the food matrix; their interpretation is not always straightforward [84], the hardness and gumminess of cheeses augmented with ripening duration (Table 5). The TP addition significantly affected the textural profile of the cheeses, resulting in increased hardness, gumminess, and chewability.

Hardness, defined as the force required to compress a sample, increased during ripening, likely as a consequence of moisture loss, whereas reduced cohesiveness can be explained by proteolytic degradation of the protein network [84]. Upon completion of the ripening period, TP-enriched cheeses exhibited higher hardness and gumminess than the control samples. These differences are consistent with the higher initial solid content and fiber incorporation associated with TP addition, as well as with the denser protein–fiber networks observed in SEM micrographs. Also, the observed textural evolution can be attributed to ripening-related matrix rearrangement and proteolytic activity, which are known to influence the mechanical properties of semi-hard cheeses [84,85]. Further studies, including compositional monitoring during storage, would be required to quantitatively link moisture loss and dry matter changes to texture development.

Creamer and Olson [86] indicate that the concentration of short peptides and free amino acids generated by casein hydrolysis escalated during ripening. These chemicals can bind free water molecules, fortify the casein matrix and enhancing both hardness levels and the cheese’s resistance to deformation.

The effort necessary to counteract the attraction between food and other surfaces is called adhesiveness. Adequate adhesiveness enhances taste and flavor release [87]. Experimental cheese treatments represented a gradual increase in adhesiveness values with the TP addition. An increase in adhesiveness was observed alongside higher fat content and lower moisture levels in TP-supplemented cheeses. This trend may be explained by changes in the cheese matrix associated with fat concentration and reduced water availability. However, adhesiveness increases in TP-supplemented cheeses throughout the ripening period [88].

Cohesiveness is a mechanical structural property that describes how a food can be deformed without breaking, resulting from the force exerted by internal bonds within the food. In the present study, the CTP7 cheese exhibited higher cohesiveness than the control sample, indicating a formulation-dependent effect of TP addition on internal structural integrity. A decrease in cohesiveness was observed for all samples over the ripening period, reflecting progressive weakening of internal bonds within the cheese matrix. Although changes in moisture content and dry matter were not measured during storage, the observed decline in cohesiveness is consistent with ripening-related matrix rearrangement (proteolysis) [89,90].

The increased gumminess and chewiness observed in TP-enhanced cheeses are consistent with the microstructural features revealed by SEM analysis. As shown in the SEM micrographs, TP-supplemented samples, particularly CTP7, exhibited a more compact and less porous protein matrix compared with the control cheese, with visible incorporation of fiber particles within the casein network. This denser structure supports stronger internal interactions, which explains the higher hardness, cohesiveness, gumminess, and chewiness values measured instrumentally. Moreover, the reduced moisture content and increased dry matter in TP-enriched cheeses contributed to closer packing of the protein–fat–fiber matrix, as evidenced by the SEM images showing a more continuous network. These structural modifications align with the textural reinforcement observed in the TP-supplemented samples and are consistent with the known effect of moisture reduction and fiber incorporation on cheese matrix rigidity. Also, Zheng et al. [91] indicated that Cheddar-type cheese exhibits high hardness but low cohesiveness and springiness owing to its low moisture content.

TP is rich in insoluble fibers, which can interact physically with the casein matrix by disrupting protein–protein interactions and acting as inert fillers within the protein network. These fibers are capable of binding and retaining water, thereby altering water distribution and reducing the mobility of the aqueous phase within the cheese structure. As a result, changes in hardness, cohesiveness, and chewiness may arise from a less homogeneous and more heterogeneous protein network, particularly at higher TP inclusion levels. Furthermore, the incorporation of fiber particles within the casein matrix can hinder the formation of a continuous protein network, leading to localized discontinuities that affect mechanical behavior. At higher TP levels, this effect may become more pronounced, contributing to increased firmness or brittleness despite overall moisture loss. These observations are consistent with previous reports on fiber-enriched cheeses and dairy matrices, where fiber–protein interactions significantly influenced textural attributes through modifications of water binding and microstructure [92,93,94].

All examined samples exhibited an increase in the values of hardness, adhesiveness, gumminess, and chewiness texture parameters during ripening. The texture analysis revealed that the incorporation of TP enhanced the textural properties of the cheeses, directly correlating with the concentration. The incorporation of grape extracts also influences the texture of the cheeses. The integration of grape extracts into the cheese matrix yields products characterized by reduced elasticity and increased hardness, attributed to the interaction of polyphenols with the cheese matrix and an enhanced loss of water due to diminished pH levels and the presence of fibers [95].

### 3.8. Mineral Profile of TP-Supplemented Cheese Samples

Table 6 indicates that the incorporation of TP led to an increase in mineral content (Ca, P, K, Mg, Zn, Na, Cu, and Fe) in the TP-enriched cheese samples relative to the control cheese. The results indicate that the incorporation of TP powder significantly modified the mineral content of the cheeses in comparison to the control samples (*p* < 0.05). The mineral composition of the control (CC) and TP powder-supplemented cheeses (CTP5 and CTP7) demonstrates a distinct pattern of elevated mineral content corresponding to increased supplementation concentration. The variations among the cheese samples for the concentrations of Mg, Fe, Na, P, K, Ca, and Zn were determined to be statistically significant (*p* < 0.05). The elevated mineral concentrations detected in the TP-supplemented cheese samples may be ascribed to the substantial mineral content found in tomato powder (Table 1). The descending order of mineral content in the CTP7 sample was calcium (Ca), potassium (K), phosphorus (P), sodium (Na), magnesium (Mg), zinc (Zn), iron (Fe), and copper (Cu).

Cheese samples are fortified with essential nutrients like calcium and phosphorus. Calcium steadily increased, rising from 271.50 mg/100 g in CC to 314.05 mg/100 g in CTP7, establishing it as the predominant mineral across all samples. The CTP7 sample, containing 7% TP powder, exhibited the highest calcium content. Phosphorus slightly increased from 228.52 mg/100 g (CC) to 244.65 mg/100 g (CTP7). The cheese containing 7% TP powder demonstrated the highest phosphorus concentration, succeeded by the cheese with 5% TP powder. Moreover, the cheese enriched with 7% TP powder exhibited the highest potassium (K) and magnesium (Mg) concentrations, whereas the cheese with 5% TP powder contained lesser quantities. Conversely, the control sample had the lowest concentrations of these minerals. Potassium significantly rose from 172.25 mg/100 g in CC to 252.73 mg/100 g in CTP7, establishing it as the second most prevalent mineral and demonstrating the most substantial improvement. Magnesium exhibited a similar favorable trend, increasing from 16.53 mg/100 g in CC to 22.85 mg/100 g in CTP7.

Statistically significant differences (*p* < 0.05) were seen in the iron, zinc, copper, and potassium levels between the TP powder samples and the control sample. The incorporation of 5% and 7% TP (CTP5 and CTP7) led to elevated concentrations of Zn, Fe, and Na compared to the control cheese. Trace elements such as zinc, iron, and copper exhibited incremental increases, with zinc rising from 3.42 mg/100 g to 4.21 mg/100 g, iron from 1.42 mg/100 g to 2.89 mg/100 g, and copper from 0.11 mg/100 g to 0.24 mg/100 g. The sodium concentration rose from 96.85 mg/100 g to 131.05 mg/100 g. CTP7 had the greatest mineral values across all enriched samples, whereas the control sample continuously displayed the lowest quantities, highlighting the nutritional enhancement potential of TP powder in cheese recipes. Table 1 indicates that freeze-dried tomato powder possesses substantial quantities of magnesium (Mg), calcium (Ca), phosphorus (P), potassium (K), and sodium (Na). Consequently, TP powder is suitable for incorporating as a food bioactive element.

El-Araby et al. [96] similarly reported that fortifying snacks with apple pomace powder and TP powder enhanced their levels of protein, ash, fiber, calcium, potassium, and phenolic chemicals. Abdelmontaleb et al. [97] discovered that soft cheese enriched with quinoa flour (0%, 1%, 2%, and 3%) possesses elevated mineral content relative to the control. Supplementation of crackers with TP resulted in a significant (*p* < 0.05) increase in protein, dietary fiber (insoluble, soluble, total), ash, minerals (Ca, Mg, P, K, Mn, Fe, Zn), total phenolic content, and antioxidant capacity [98].

### 3.9. Microstructure Analysis of TP-Supplemented Cheese Samples

**Figure 4:**
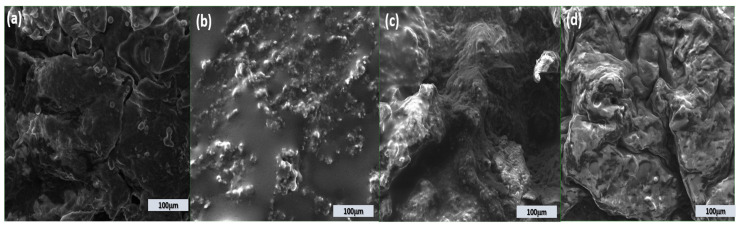
SEM micrographs of TP powder (**a**) control semi-hard cheese ((**b**) CC) TP-supplemented cheese ((**c**) CTP5 and (**d**) CTP7).

Figure 4 illustrates the microstructure of the TP, cheese sample, and TP-Supplemented Cheese sample as observed using electron microscopy. The microstructure of the control cheese distinctly differs from that of the other experimental cheese varieties; the microstructure of the control cheese was more open than that of the other enriched cheeses.

The integration of TP can be described as a homogeneous protein uniformly embedded and distributed inside the cheese matrix, in contrast to the control sample. The study revealed that the protein matrix included uniformly distributed larger fat globules throughout the cheese structure compared to the control sample. The microstructural characteristics of enhanced cheeses align with those of processed cheese [99].

The cheeses display varying microstructures based on the incorporation of fibers. At a magnification of 500× (100 μm), the semi-hard sample exhibited a homogeneous look devoid of granules. Moreover, the characteristic protein clumps typically seen in cheese were observable. In experimental cheeses, fiber particles are observable at 500× magnification (CTP5 and CTP7).

**Figure 5:**
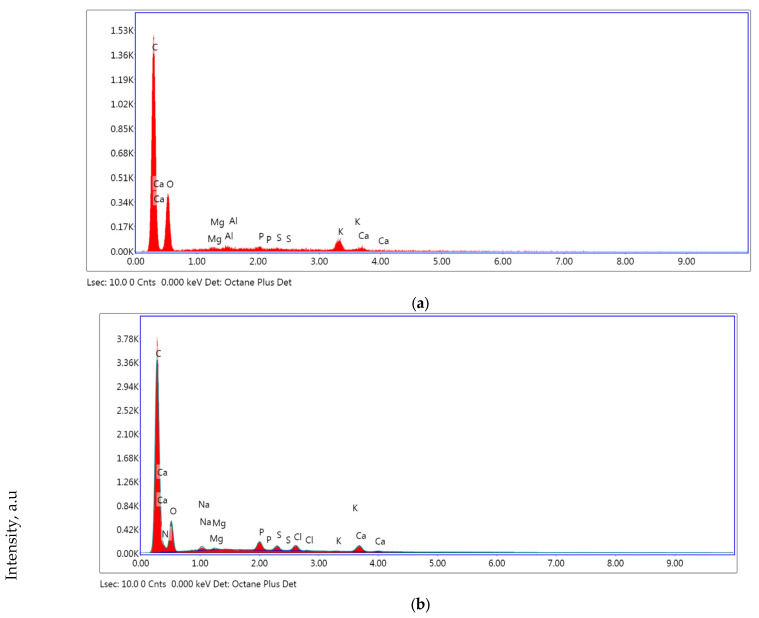

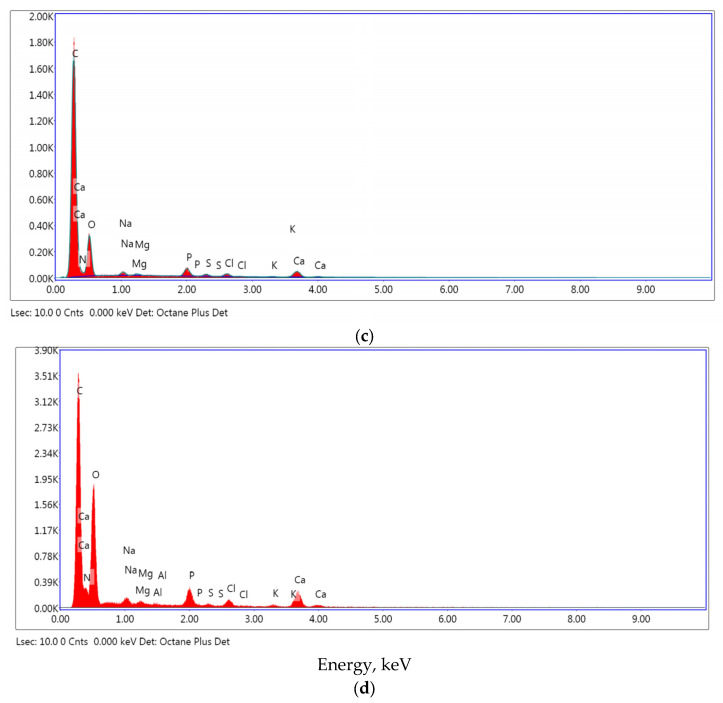
SEM-EDX spectrum of TP powder (**a**) control semi-hard cheese ((**b**) CC) TP-supplemented cheese ((**c**) CTP5 and (**d**) CTP7).

Figure 5 illustrates the EDX spectrum of the analyzed product. The EDX spectrum of the samples indicated the presence of alkali ions (potassium, sodium) and alkaline earth ions (magnesium, calcium) on the surface, consistent with the results in Table 1.

The protein aggregates network in the fiber-enriched samples exhibits a structure different from that of CC cheese. An open structure featuring bigger particles was observed. Comparable findings were seen by [100].

The existence of broad and profound aggregates in the supplemented samples signified the occurrence of proteolysis, reconfiguration of the protein matrix, and an enhancement in the firmness of the semi-hard cheese texture. During proteolysis, intact caseins, which facilitate network formation, decompose into small and medium-sized peptides and free amino acids, which are subsequently released into the serum part of the cheese [101]. The documented increase in cheese hardness, as indicated in Table 4, substantiated this. This study found that the reduced pH of TP-enriched semi-hard cheese samples facilitated sub-micelles’ disintegration into non-linear casein strands, ultimately resulting in the aggregation of the protein matrix. This was corroborated by Tunick [87].

The microstructure of the cheese samples exhibited variations, with CTP5 and CTP7 cheeses displaying a heterogeneous composition defined by a tightly interconnected network. Casein micelles were more prominent in the designated samples than in the control cheese. The results of CTP7 demonstrated a design characterised by increased density and a more compact structure. Cheeses, including TP powder, exhibited enhanced cross-linking and linkages. The increased density of the matrix renders these cheese samples firmer. The SEM analysis indicated that cheese samples with elevated TP powder concentrations contained more protein aggregates in their matrices than the control group.

Due to the elevated TP content, CTP7 cheese casein clusters exhibited greater thickness and density than other samples. Due to its dietary fibre content, TP powder enhances the texture and structure of cheese. The enhanced hardness of CTP7 may be attributed to its denser structure. The results align with the observations of Alqattan et al. [102], who noted that including hydrocolloids and date pit powder in spreadable semi-hard cheese and processed cheese positively influenced the texture of the products.

### 3.10. Sensorial Analysis of TP-Supplemented Cheese Samples

**Figure 6:**
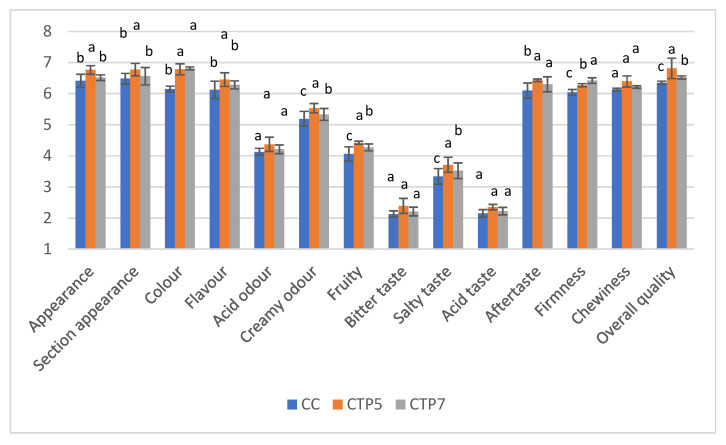
Comparative diagram of the sensory attributes specific to TP-Supplemented cheeses: CC cheese without powder addition; CTP5 and CTP7 cheese with 5 and 7% TP (averages with different letters “a”, “b”, “c” in the columns signify statistically significant differences (*p* < 0.05)).

The sensory assessment of the produced cheeses was performed on a seven-point hedonic scale. Figure 6 displays the average ratings obtained from the sensory evaluation. Sensory attributes involve the assessment of: visual appearance, sectional appearance, colour, flavour, acid odour, creamy odour, fruity, bitter taste, salty taste, acid taste, aftertaste, firmness, chewiness, and overall quality. Semi-hard cheese features a smooth rind, a yellowish hue, rare fermentation meshes, an elastic texture, and uniformity throughout its composition.

The panelists determined that the TP-supplemented cheese was denser than the control. TP-supplemented cheese had a higher smell strength and saltiness in comparison to the control. The incorporation of tomato powder led to a major change in color, with the cheese displaying a more vivid orange-red shade. The color intensity increased with the increased proportion of tomato powder, with the CTP7 sample (7% TP) exhibiting a deeper and more prominent hue than the CTP5 sample (5% TP). Cheeses treated with 5% and 7% TP powder had a smooth and firm texture. The CTP7 sample (7% TP) had a slightly harder and denser consistency, while the CTP5 sample (5% TP) was regarded as creamier, with a slight enhancement in elasticity.

The sensory analysis results corroborated several findings from the texture analysis, indicating that TP-supplemented cheese was firmer than the control sample. TP-supplemented cheeses had a higher smell intensity and saltiness than the control cheeses. The odor intensity of cheese was affected by the degree of proteolytic and lipolytic processes [103]. Additionally, the perception of saltiness may be influenced by proteolysis, as the final products of these processes, such as amino acids, can enhance saltiness [104]. Attributes such as increased hardness or enhanced taste and flavor may elevate the product’s attractiveness among cheese consumers who like flavourful varieties, rendering the implemented supplementation technique appropriate for the effective valorization of the TP.

The cheese with a moderate addition of TP garnered positive evaluations from panelists about its appearance, color, flavor, taste, and general acceptability. The flavor of the enhanced cheese samples was enriched with a subtle yet distinct tomato scent. The CTP7 sample (7% TP) exhibited a more robust tomato aroma, while the CTP5 sample (5% TP) had a milder, more delicate odor. Both samples retained the characteristic cheese aroma, with the tomato scent enhancing the overall sensory profile.

Both TP-supplemented cheese samples showed a balanced flavor, with the tomato powder adding a mild, savory note that complemented the cheese’s natural taste. The CTP7 sample (7% TP) had a more pronounced tomato flavor, which was well-received by some panelists, though others found the CTP5 sample (5% TP) to be more harmonious and less overpowering. The aftertaste of the cheese was predominantly agreeable, featuring a persistent, mildly acidic nuance from the tomato powder. The 7% tomato powder cheese sample exhibited a more pronounced aftertaste, favored by some panelists, but others deemed the CTP5 sample (5% TP) to possess a more nuanced and pleasant finish.

The panelists favorably evaluated both cheese samples enhanced with tomato powder, with the CTP5 sample (5% TP) receiving a marginally higher overall acceptability rating owing to its more harmonious flavor and creamier texture. The CTP7 sample (7% TP) was characterized by an intense, vivid color (Figure 6); however, some panelists perceived its flavor as overly strong, with a slight bitter aftertaste.

Overall assessment scores indicated that the semi-hard cheese was more acceptable with 5% of TP than the cheese sample with 7% of TP, maybe due to the use of 7% TP producing a slightly bitter aftertaste. Also, larger, demographically diverse consumer studies are required to confirm the acceptance and market potential of TP-enriched cheeses.

**Figure 7:**
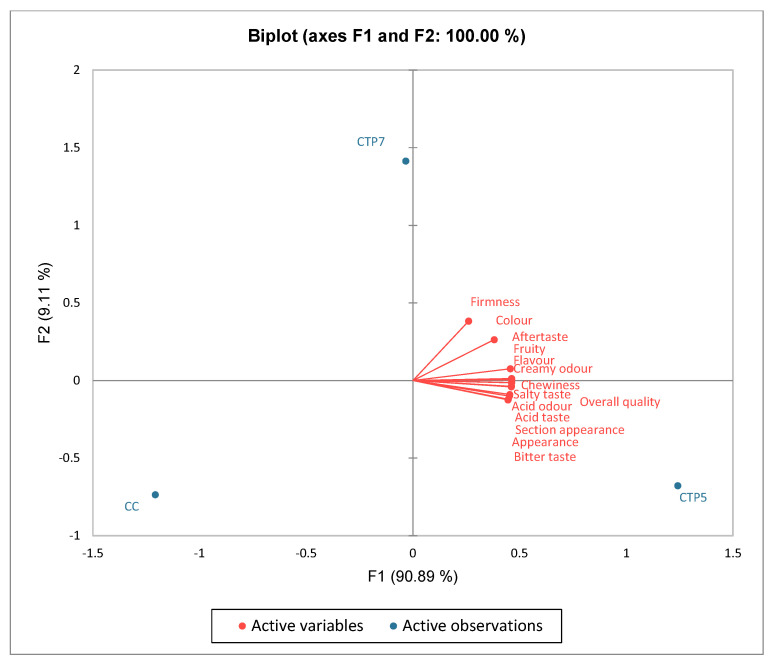
Principal Component Analysis (PCA) biplot of the position of the three cheeses (CC, CTP5, and CTP7) for sensory attributes evaluation.

Figure 7 presents a PCA biplot that visually illustrates the locations of the three cheese samples (plain compared to those enhanced with TP) according to the sensory qualities outlined in Figure 6. The initial principal component (PC1) represented 90.89% of the variation and included the two cheese samples with TP. The second component constituted 9.11% of the total and comprised of plain cheese. Consequently, the two axes represented 100.00% of the total variation. The firmness, color, aftertaste, fruity, flavor, and creamy odor highly correlated on the first axis, F1. Likewise, the characteristics of chewiness, salty taste, acid taste, acid odor, bitter taste, appearance, section appearance, and overall quality were also identified as having a positive correlation within the same quadrant on the first axis, F1. The plain cheese was considered neutral since all sensory attributes were exclusively linked to the same axis, F1. The biplot analysis facilitates accurately positioning the three cheese samples according to their sensory attributes. It facilitates the distinction of sensory properties via positive correlations between sensory attributes and cheese samples.

The experimental cheeses exhibited intricate scents and a more pronounced aroma. The panelists’ total judgment indicated that cheese enhanced with 5% TP powder received the highest appreciation from the assessors. Although the 7% TP cheese exhibited higher phytochemical content and antioxidant activity, its reduced sensory acceptability, mainly due to increased bitterness associated with elevated polyphenol levels, limits its immediate consumer appeal. In contrast, the 5% TP formulation achieved a more favorable balance between functional enhancement and sensory quality, suggesting greater potential for consumer acceptance.

**Figure 8:**
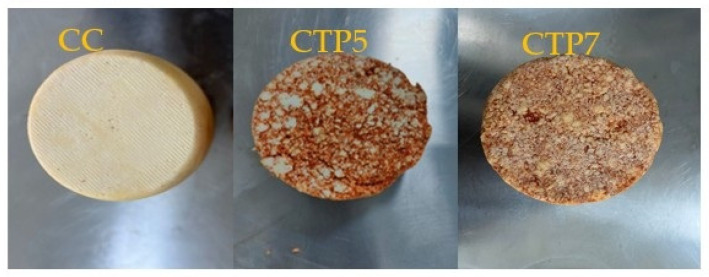
Cheese samples with different percentages of TP: CC (control), 5% (CTP5), and 7% (CTP7).

Figure 8 illustrates the appearance of cheese samples formulated with increasing TP concentrations: CC (control), 5% TP (CTP5), and 7% TP (CTP7). Adopting a food product generally indicates its real use, including acquisition and consumption. Incorporating plant powders or extracts into fortified goods generally enhances their overall acceptability, particularly regarding flavour, owing to appealing aromatic components and phenolic chemicals [16,105]. Rizk et al. [25] demonstrated the potential of improving ice cream properties by incorporating natural colorants and antioxidants derived from TP. The study highlighted that adding TP significantly enhanced the product’s functional properties, including higher radical scavenging activity, which indicates improved antioxidant capacity. Sensory evaluation revealed that the ice cream scored higher in critical attributes such as flavor, body and texture, melting quality, and color than conventional formulations. These findings underscore the value of utilizing agro-industrial by-products like TP to enhance ice cream’s nutritional and sensory quality, aligning with current trends in functional and sustainable food product development [25].

In addition, Kaur et al. [64] explored the use of food waste and by-products to enhance the quality of ice cream. They utilized lycopene crystals extracted from TP as an antioxidant to mitigate off-flavors, off-odors, and color changes while storing dairy products. The study found that this fortification improved the antioxidant activity and extended the shelf life of ice cream while maintaining its sensory acceptability. Nonetheless, a recent investigation indicated that extruded snacks with 10% TP exhibited the highest marks regarding physical and sensory attributes [64]. This indicates that the degree of TP fortification and the acceptance of the final product differ based on the food matrix.

## 4. Conclusions

The results highlighted the importance of TP extract as a significant source of bioactive chemicals, including minerals, lycopene, and phenolic substances with remarkable antioxidant capabilities. This enrichment technique enabled the creation of a bovine semi-hard cheese with innovative functional, physicochemical, and sensory properties. The results confirm that TP powder represents a valuable source of bioactive-related compounds and dietary fiber that can be incorporated into semi-hard cheese, contributing to enhanced antioxidant-related indices and product differentiation.

However, the study also demonstrates a clear trade-off between functional enrichment and sensory quality. While higher TP addition (7%) resulted in greater phytochemical-related values, it adversely affected sensory acceptability due to bitterness. In contrast, the 5% TP formulation achieved a more favorable balance between functional enhancement, texture, and sensory acceptance, indicating greater potential for consumer-oriented applications. These findings highlight the importance of optimizing inclusion levels to maximize nutritional benefits without compromising sensory quality.

Future studies should focus on addressing the limitations identified in this work. In particular, trained sensory panel evaluations combined with large-scale consumer acceptance tests are needed to establish the maximum acceptable level of TP incorporation in semi-hard cheese. Given the observed bitterness at higher inclusion levels, formulation or processing strategies (e.g., encapsulation of polyphenols) should be investigated to improve sensory quality. In addition, in vitro digestion and bioaccessibility studies are required to evaluate the release and bioavailability of lycopene and other bioactive compounds from the cheese matrix. Further research should also examine storage stability, lipid oxidation indicators, and microbiological safety to support potential industrial-scale application.

Effectively incorporating TP into semi-hard cheese boosts its nutritional profile and fosters the sustainable utilization of agricultural by-products, aiding the food industry’s transition to more sustainable methods.

## Figures and Tables

**Table 1:** Global phytochemical characterization of TP.

Parameter	TP
Total Carotenoids, µg/g dw	1030.81 ± 0.53
Licopen, µg/g dw	561.25 ± 0.32
Total Flavonoids, mg CE/100 g dw	112.18 ± 1.38
Total Polyphenols, mg GAE/100 g dw	344.38 ± 2.06
Antioxidant activity, µmol TE/g dw	1431.60 ± 5.84
Inhibition, %	94.58 ± 0.39
L*	48.43 ± 0.15
a*	26.82 ± 0.12
b*	36.28 ± 0.05
Chroma	45.12 ± 0.13
Hue Angle	0.93 ± 0.03
Moisture (%)	10.52 ± 0.04
Ash (%)	4.80 ± 0.02
Protein (%)	11.45 ± 0.06
Carbohydrates (%)	67.09 ± 0.14
Fat (%)	6.14 ± 0.03
Total dietary fiber (%)	46.10 ± 0.02
Calcium (Ca, mg/100 g)	145.02 ± 0.02
Phosphorus (P, mg/100 g)	14.23 ± 0.07
Potassium (K, mg/100 g)	49.95 ± 0.08
Magnesium (Mg, mg/100 g)	141.25 ± 0.14
Zinc (Zn, mg/100 g)	3.66 ± 0.03
Iron (Fe, mg/100 g)	10.29 ± 0.05
Sodium (Na, mg/100 g)	23.85 ± 0.09
Copper (Cu, mg/100 g)	1.54 ± 0.02

**Table 2:** Phytochemical characteristics, antioxidant activity, and stability during 30 days of storage for the control and TP-supplemented cheese samples.

Phytochemical Characteristics	Storage Time, (Days)	CC	CTP5	CTP7
Total Carotenoids, mg/100 g fresh weight	0	-	90.05 ± 0.86 ^bB^	140.41 ± 1.37 ^bA^
	30	-	120.07 ± 0.94 ^aB^	180.37 ± 1.52 ^aA^
Total Lycopene, mg/100 g fresh weight	0	-	6.07 ± 0.71 ^bB^	11.26 ± 1.08 ^bA^
	30	-	9.14 ± 0.90 ^aB^	15.31 ± 1.29 ^aA^
Total Polyphenols, mg GAE/100 g fresh weight	0	84.66 ± 1.71 ^bC^	101.50 ± 2.03 ^bB^	123.66 ± 2.39 ^bA^
	30	116.19 ± 2.11 ^aC^	132.08 ± 2.59 ^aB^	155.21 ± 2.74 ^aA^
Total Flavonoids, mg CE/100 g fresh weight	0	47.41 ± 1.51 ^bC^	76.08 ± 2.14 ^bB^	98.25 ± 2.76 ^bA^
	30	72.26 ± 1.81 ^aC^	102.37 ± 2.69 ^aB^	124.49 ± 2.96 ^aA^
Antioxidant activity, µmol TE/g fresh weight	0	215.24 ± 4.19 ^bC^	566.89 ± 9.37 ^bB^	831.71 ± 10.04 ^bA^
	30	232.38 ± 4.22 ^aC^	597.78 ± 9.52 ^aB^	870.32 ± 10.21 ^aA^

Means marked with distinct superscript lowercase letters indicate a statistically significant difference (*p* < 0.05) between each investigated phytochemical and storage time. Means with various superscript uppercase letters (*p* < 0.05) indicate a significant difference between each investigated phytochemical and sample variant.

**Table 3:** Physico-chemical characteristics of control and supplemented cheese samples.

Physical-Chemical Characteristics	CC	CTP5	CTP7
Dry matter, %	51.19 ± 0 19 ^c^	53.75 ± 0.22 ^b^	55.87 ± 0.26 ^a^
pH	5.18 ± 0.15 ^a^	5.10 ± 0.13 ^a^	5.06 ± 0.10 ^a^
Fat, %	26.40 ± 0.18 ^c^	27.69 ± 0.20 ^b^	28.75 ± 0.31 ^a^
Protein, %	14.90 ± 0.23 ^c^	15.29 ± 0.29 ^b^	16.43 ± 0.32 ^a^
Carbohydrates, %	18.88 ± 0.12 ^a^	15.12 ± 0.17 ^b^	11.97 ± 0.14 ^c^
Fiber, %	0.00 ± 0.00 ^c^	3.62 ± 0.14 ^b^	5.44 ± 0.18 ^a^
Moisture, %	48.81 ± 0.32 ^a^	46.25 ± 0.36 ^b^	44.13 ± 0.39 ^c^
Salt, %	1.02 ± 0.09 ^a^	0.94 ± 0.08 ^a^	0.78 ± 0.07 ^a^
Ash, %	2.46 ± 0.19 ^b^	2.55 ± 0.21 ^ab^	2.62 ± 0.25 ^a^
Energetic value, Kcal/100 g	372.72 ± 0.15 ^c^	378.09 ± 0.17 ^b^	383.23 ± 0.19 ^a^
KJ/100 g	1557.97 ± 0.15 ^c^	1580.42 ± 0.17 ^b^	1601.90 ± 0.19 ^a^

For every physicochemical parameter and sample, means that do not have the same superscript lowercase letter are substantially different at a significance level of *p* < 0.05.

**Table 4:** Colorimetric parameters of control and TP-supplemented cheese samples during storage for 30 days.

Samples	Storage Time (Day)	L*	a*	b*	Chroma	H*	ΔE
CC	0	85.55 ± 0.39 ^aA^	−3.27 ± 0.08 ^aC^	29.09 ± 0.29 ^aC^	29.27 ± 0.11 ^aC^	178.54 ± 0.03 ^aA^	-
	30	82.74 ± 0.35 ^bA^	−2.88 ± 0.07 ^bC^	31.38 ± 0.27 ^bC^	31.51 ± 0.13 ^bC^	178.52 ± 0.02 ^aA^	-
CTP5	0	62.95 ± 0.32 ^aB^	18.61 ± 0.05 ^aB^	31.98 ± 0.23 ^aB^	37.01 ± 0.12 ^aB^	1.04 ± 0.02 ^aB^	31.59 ± 0.04 ^aB^
	30	59.92 ± 0.30 ^bB^	20.19 ± 0.13 ^bB^	35.41 ± 0.26 ^bB^	40.76 ± 0.18 ^bB^	1.05 ± 0.02 ^aB^	36.48 ± 0.06 ^bB^
CTP7	0	58.51 ± 0.36 ^aC^	20.67 ± 0.11 ^aA^	34.29 ± 0.29 ^aA^	40.04 ± 0.13 ^aA^	1.03 ± 0.04 ^aB^	32.70 ± 0.04 ^aA^
	30	55.86 ± 0.31 ^bC^	22.81 ± 0.19 ^bA^	37.35 ± 0.30 ^bA^	43.76 ± 0.14 ^bA^	1.02 ± 0.02 ^aB^	37.66 ± 0.05 ^bA^

Color parameter variation over storage is highlighted by superscript lowercase letters. The color variations among the samples are indicated by superscript uppercase letters. Values that do not share a lower/uppercase letter differ significantly (*p* < 0.05).

**Table 5:** Textural parameters of the control and TP-supplemented cheeses during storage for 30 days.

Parameter	Storage Period (Day)	CC	CTP5	CTP7
Hardness, N	0	7.90 ± 0.16 ^cB^	15.71 ± 0.20 ^bB^	20.15 ± 0.27 ^aB^
	30	10.81 ± 0.19 ^cA^	19.64 ± 0.25 ^bA^	24.85 ± 0.31 ^aA^
Adhesiveness, mJ	0	0.20 ± 0.09 ^cA^	0.41 ± 0.13 ^bB^	0.63 ± 0.17 ^aB^
	30	0.29 ± 0.10 ^cA^	0.55 ± 0.14 ^bA^	0.76 ± 0.18 ^aA^
Cohesiveness -	0	0.59 ± 0.15 ^cA^	0.78 ± 0.18 ^bA^	0.99 ± 0.22 ^aA^
	30	0.48 ± 0.11 ^cB^	0.67 ± 0.16 ^bB^	0.83 ± 0.20 ^aB^
Elasticity, -	0	0.68 ± 0.14 ^bA^	0.72 ± 0.17 ^abA^	0.87 ± 0.23 ^aA^
	30	0.52 ± 0.09 ^bB^	0.63 ± 0.13 ^abB^	0.74 ± 0.18 ^aB^
Gumminess, N	0	7.12 ± 0.23 ^cB^	16.89 ± 0.25 ^bB^	19.35 ± 0.27 ^aB^
	30	9.32 ± 0.24 ^cA^	18.41 ± 0.26 ^bA^	21.51 ± 0.29 ^aA^
Chewability, -	0	4.93 ± 0.15 ^cB^	11.97 ± 0.17 ^bB^	16.88 ± 0.24 ^aB^
	30	6.54 ± 0.17 ^cA^	13.68 ± 0.22 ^bA^	18.79 ± 0.26 ^aA^

Values with the same superscript lowercase letter for each textural parameter and sample are not statistically different in terms of time at *p* > 0.05. Textural parameters with the same superscript uppercase letter for each storage time are not statistically different at a significance level of *p* > 0.05.

**Table 6:** Mineral composition of the control and TP-Supplemented cheeses samples.

Parameter	CC	CTP5	CTP7
Calcium (Ca, mg/100 g)	271.50 ± 1.02 ^c^	297.65 ± 1.05 ^b^	314.05 ± 1.09 ^a^
Phosphorus (P, mg/100 g)	228.52 ± 0.77 ^c^	236.51 ± 0.81 ^b^	244.65 ± 0.92 ^a^
Potassium (K, mg/100 g)	172.25 ± 0.91 ^c^	241.47 ± 1.04 ^b^	252.73 ± 1.24 ^a^
Magnesium (Mg, mg/100 g)	16.53 ± 0.21 ^c^	19.79± 0.29 ^b^	22.85 ± 0.35 ^a^
Zinc (Zn, mg/100 g)	3.42 ± 0.11 ^c^	3.84 ± 0.18 ^b^	4.21 ± 0.20 ^a^
Iron (Fe, mg/100 g)	1.42 ± 0.09 ^c^	2.19 ± 0.16 ^b^	2.89 ± 0.19 ^a^
Sodium (Na, mg/100 g)	96.85 ± 0.38 ^c^	116.85 ± 0.42 ^b^	131.05 ± 0.59 ^a^
Copper (Cu, mg/100 g)	0.11 ± 0.04 ^b^	0.20 ± 0.08 ^a^	0.24 ± 0.11 ^a^

Superscripts with different letters within a row are significantly (*p* < 0.05) different.

## Data Availability

The original contributions presented in this study are included in the article. Further inquiries can be directed to the corresponding author.
